# Stauffer Syndrome: A Comprehensive Review of the Icteric Variant of the Syndrome

**DOI:** 10.7759/cureus.6032

**Published:** 2019-10-30

**Authors:** Neha Sharma, Umar Darr, Amir Darr, Gagan Sood

**Affiliations:** 1 Internal Medicine, Abdominal and Liver Transplantation, Baylor College of Medicine, Houston, USA; 2 Internal Medicine, Houston Methodist, Houston, USA; 3 Medicine, University of Limerick, Limerick, IRL; 4 Internal Medicine: Gastroenterology, Abdominal Transplantation, Baylor College of Medicine, Houston, USA

**Keywords:** stauffer syndrome, renal cell carcinoma, paraneoplastic syndrome, paraneoplastic hepatopathy

## Abstract

Stauffer syndrome is a rare paraneoplastic syndrome classically associated with renal cell carcinoma (RCC). This association gave it the historical name of nonmetastatic nephrogenic hepatic dysfunction syndrome without jaundice. It is a syndrome of unclear pathophysiology characterized by a reversible anicteric elevation of liver enzymes, alkaline phosphatase, erythrocyte sedimentation rate (ESR), thrombocytosis, prolongation of prothrombin time, and hepatosplenomegaly in the absence of direct hepatobiliary obstruction or jaundice. A rare atypical variant of this syndrome with jaundice has been recently described in the literature. Thus, it is important to consider both these variants of Stauffer syndrome in the differential diagnosis of unexplained cholestasis in the absence of hepatic metastasis. This may allow early recognition and treatment of an occult malignancy. Herein, we present a comprehensive review of the literature available on the icteric variant of the Stauffer syndrome, outlining its association with various malignancies and the diagnostic challenges it poses. The objective of this review is to help clinicians in its early diagnosis and management.

## Introduction and background

Stauffer syndrome, also known as Block‐Stauffer‐Rothmand's Syndrome, Thomson‐Rothmand's Syndrome, ‘nephrogenous hepatosplenomegaly’ or ‘nephrogenous hepatic dysfunction’ was first described by an American gastroenterologist Maurice H. Stauffer in 1961. He noted that patients suffering from renal cell carcinoma (RCC) had abnormal liver function tests, hepatosplenomegaly, and nonspecific hepatitis type histologic changes in the absence of hepatic metastasis. Interestingly, tumor removal led to the reversal of these abnormalities [1]. This led to the conclusion that hepatic dysfunction in these patients was of a paraneoplastic nature. This syndrome has been classically described in relation to the early stages of renal cell cancer, but over time, literature has emerged supporting its association with other cancers including bronchogenic carcinoma, bladder cancer, pancreatic cancer, metastatic prostate adenocarcinoma, soft tissue sarcoma, and malignant lymphoproliferative diseases.

## Review

The term paraneoplastic syndrome refers to a constellation of systemic signs and symptoms that are secondary to malignancy and does not include the sequelae of either direct tumor extension or metastasis. They are believed to be the result of the following processes: i) production of humoral substances by tumor cells, ii) production of humoral factors in response to malignancy by benign tissues, or iii) via immune system modulation [1].

A review of all the available data, published in the English language, yielded more than 100 cases of Stauffer syndrome since it was first described in 1961. Most cases are described in association with RCC, with an incidence of 3%-6% among these patients [2]. Prostate cancer was a close second followed by soft tissue sarcomas [3], pancreatic cancer, bladder cancer [4], malignant lymphoproliferative diseases [5], and isolated cases with bronchogenic carcinoma [6], gastrointestinal carcinoid [7], and one case report with a thrombocytopenia variant [8]. A vast majority of cases are of classical nature, and only a handful of jaundice variants have been reported to date, as detailed in Table 1. In 1997, the first two cases of jaundice variant were described by Dourakis et al. in association with RCC [9]. These variant cases can present with hyperbilirubinemia, urinary hyperpigmentation, hepatosplenomegaly, jaundice, and/or pruritus along with the classical findings of elevated liver function tests (LFTs), erythrocyte sedimentation rate (ESR), alpha-2 globulin, platelets, and prothrombin time [2].

**Table 1:** Published case reports of icteric variant of Stauffer syndrome with corresponding type of cancer.

Malignancy associated with Stauffer syndrome jaundice variant	References (Author, Year of publication, Ref. )
Renal cell carcinoma	Mazokopakis and Papadakis (2007) [2], Dourakis et al. (1997) [9], Morla et al. (2006) [6], Akbulut et al. (2014) [10], Giannakos et al. (2005) [11], Sarf et al. (2003) [12], Tomadoni et al. (2010) [13], Fernandez and de Ávila (2012) [14], Jangouk and Hashash (2014) [15], Puga et al. (2015) [16], Ates et al. (2015) [8], Current report (2019)
Prostate carcinoma	Kato et al. (2014) [17], Kuramoto et al. (2013) [18], Hinostroza-Yanahuaya et al. (2013) [19], Bhangoo et al. (2018) [20], Ravindranathan et al. (2018) [21], Gokcen et al. (2019) [22], Romasovs et al. (2019) [23]
Pancreatic cancer	Harris and Saif (2017) [4]
Gastrointestinal carcinoid	Mehta (2017) [7]

Paraneoplastic hepatopathy typically consists of unexplained elevation in liver enzymes, with or without jaundice, without the evidence of anatomic obstruction of bile flow, infectious etiology, or neoplastic involvement of the liver or bile ducts. The resolution of hepatopathy is noted after successful chemotherapy or surgical treatment of the primary tumor. One theory explaining the mechanism of paraneoplastic liver dysfunction suggests the release of either a humoral substance or lysosomal enzymes from the tumor, with distant effects on the liver and hematopoietic system. An increase in lysosomal enzyme activity in liver cells of patients with renal carcinoma has supported this theory. Additional speculated mechanisms include generalized hepatic hypervascularity seen on angiography, hepatic amyloid deposition, nonspecific focal periportal inflammation on liver biopsy, or an autoimmune phenomenon directed against a tumor antigen which cross-reacts with a liver protein involved in hepatic bilirubin transport [24]. Elevated IL-6 appears to be a recurring theme of Stauffer syndrome with its presence reported in a majority of cases in the literature. Blay et al. documented that patients with RCC and elevated alkaline phosphatase have significantly higher levels of serum IL-6 [15, 25]. Bhangoo et al. suggested the association of IL-6 and cholestasis may be mediated by a systemic inflammation as laboratory markers including C-reactive protein (CRP) and haptoglobin are elevated in these patients. These pro-inflammatory cytokines inhibit expression of the hepatobiliary transporter gene expression, possibly explaining impaired biliary outflow [20]. A study conducted by Karakolio et al. found that treatment with anti-interleukin 6 monoclonal antibody reversed most of the biochemical abnormalities of patients with Stauffer syndrome [5]. Hence, it can be concluded that IL-6 plays a central role in the pathogenesis of Stauffer syndrome. This also explains why patients with elevated levels of serum IL‐6 and CRP have elevated levels of haptoglobin, bilirubin, gamma glutamyl‐transferase, platelets, polymorphonuclear neutrophils (PMN), and alkaline phosphatase. It is also seen that in patients with serum IL‐6 over 80 pg/mL (normal: < 5 pg/mL), hemoglobin levels are found to be significantly lower than in patients with low/undetectable serum IL-6 levels [11]. The exact pathophysiology is yet to be determined [15].

Elevated alkaline phosphatase is the most common laboratory finding seen in approximately 90% of Stauffer syndrome cases. Elevated transaminases and hyperbilirubinemia are seen in 21% and 15% of cases, respectively [15]. A typical patient will have an anicteric presentation but an atypical variant with jaundice has also been described in the literature [1]. Other laboratory abnormalities include elevation of ESR, alpha-2-globulin, gamma-glutamyl transferase, thrombocytosis, and prolongation of prothrombin time [1, 6]. With these findings, patients are often worked up for a hepatic source. Investigations include serological tests for viral hepatitis A, B, C, δ, cytomegalovirus (CMV), Epstein‐Barr virus, Herpes virus, HIV, leptospirosis, and immunological assays for autoimmune diseases (antinuclear antibody, antimitochondrial antibody, anti-smooth muscle antibody, anti dsDNA, anti ssDNA, anti-histone antibody, p‐ANCA, c‐ANCA, Ra‐factor) [11]. In addition, iron studies, serum ceruloplasmin level, and alpha-fetoprotein levels are also performed to rule out metabolic causes and primary hepatic cancer [6]. Upper abdominal ultrasound is usually within normal limits or can show a marginally increased echogenicity and size of the liver parenchyma, without any dilatation of intra‐ or extra‐hepatic bile ducts or any other sign of localized lesions. Endoscopic retrograde cholangiopancreatography (ERCP) and magnetic resonance cholangiopancreatography (MRCP) are often within normal limits [25]. Liver biopsy will reveal cholestasis with occasional nonspecific infiltration by neutrophils, lymphocytes, monocytes, and rarely granulomas [4, 11]. With such an extensive workup often unrevealing of any evidence of known hepatic pathology, physicians must consider the possibility of cholestasis as a paraneoplastic presentation and should directly focus on unmasking an underlying occult malignancy. Ultrasonography can have certain pitfalls in detecting RCC and isoechoic RCC could be missed in routine abdominal ultrasonography [26]. CT abdomen or MRI is a more sensitive modality to locate a tumor. 

The treatment of Stauffer syndrome is directed towards the treatment of underlying malignancy. Surgical removal of an otherwise local cancer via nephrectomy leads to dramatic and complete resolution of hepatic abnormalities [4]. Recurrence of liver enzyme abnormalities in conjunction with tumor recurrence has been reported as well. However, this syndrome must be differentiated from jaundice caused by metastatic infiltration of the liver, which carries a considerably worse prognosis [5].

## Conclusions

Stauffer syndrome is an uncommon but important paraneoplastic manifestation of RCC and other malignancies. Clinicians should be aware of RCC's propensity to present as a broad spectrum of nonrenal manifestations and should have a high index of suspicion when encountered with unexplained liver abnormalities with or without jaundice. Appropriate imaging studies should be performed to make an early diagnosis and increase the likelihood of operative success. Treatment of underlying malignancy appears to resolve hepatic abnormalities. 
